# Interleukin-23 Drives Intestinal Inflammation through Direct Activity on T Cells

**DOI:** 10.1016/j.immuni.2010.08.010

**Published:** 2010-08-27

**Authors:** Philip P. Ahern, Chris Schiering, Sofia Buonocore, Mandy J. McGeachy, Dan J. Cua, Kevin J. Maloy, Fiona Powrie

**Affiliations:** 1 Sir William Dunn School of Pathology, University of Oxford, Oxford, OX1 3RE, England, UK; 2 Translational Gastroenterology Unit, Experimental Medicine Division, Nuffield Department of Clinical Medicine, John Radcliffe Hospital, Oxford, OX39DU, England, UK; 3 Merck Research Laboratories, Palo Alto, CA 94304, USA

**Keywords:** CELLIMMUNO

## Abstract

Mutations in the *IL23R* gene are linked to inflammatory bowel disease susceptibility. Experimental models have shown that interleukin-23 (IL-23) orchestrates innate and T cell-dependent colitis; however, the cell populations it acts on to induce intestinal immune pathology are unknown. Here, using *Il23r^−/−^* T cells, we demonstrated that T cell reactivity to IL-23 was critical for development of intestinal pathology, but not for systemic inflammation. Through direct signaling into T cells, IL-23 drove intestinal T cell proliferation, promoted intestinal Th17 cell accumulation, and enhanced the emergence of an IL-17A^+^IFN-γ^+^ population of T cells. Furthermore, IL-23R signaling in intestinal T cells suppressed the differentiation of Foxp3^+^ cells and T cell IL-10 production. Although *Il23r^−/−^* T cells displayed unimpaired Th1 cell differentiation, these cells showed impaired proliferation and failed to accumulate in the intestine. Together, these results highlight the multiple functions of IL-23 signaling in T cells that contribute to its colitogenic activity.

## Graphical abstract

**Graphical Abstract:**

**Figure:**
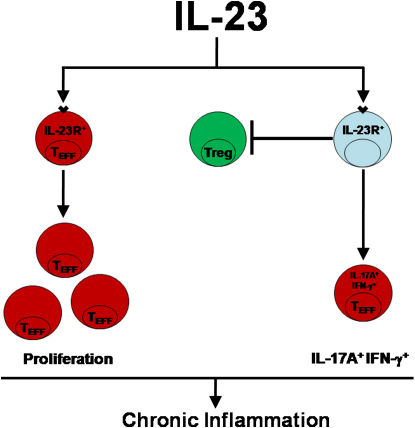

**Highlights:**

► IL -23 signaling in T cells drives intestinal but not systemic inflammation ► IL-23 signaling in T cells promotes Th17 and Th1 cell proliferation in the colon ► IL-23 signaling in T cells promotes the emergence of IL-17A^+^IFN-γ^+^ T cells ► IL-23 signaling in T cells inhibits iTreg cell accumulation and activity in the colon

## Introduction

The inflammatory bowel diseases (IBDs), comprising Crohn's Disease (CD) and ulcerative colitis (UC), are severe inflammatory disorders of the gastrointestinal tract whose incidence are on the increase (Xavier and Podolsky, 2007). Current therapeutic targeting of proinflammatory cytokines such as TNF-α has proved to be effective, but can lead to deleterious side effects and approximately one-third of patients fail to respond (Podolsky, 2002). These results highlight the need for the identification of new and more specific therapeutic targets for the treatment of IBDs.

Interleukin-23 (IL-23), a heterodimeric cytokine comprising IL-12p40 and IL-23p19 (Oppmann et al., 2000), is now well documented to be critical in the pathogenesis of a number of murine models of autoimmune and inflammatory conditions such as experimental autoimmune encephalomyelitis (EAE), collagen induced arthritis (CIA), and intestinal inflammation (Cua et al., 2003; Hue et al., 2006; Kullberg et al., 2006; Murphy et al., 2003; Uhlig et al., 2006b; Yen et al., 2006). The activity of IL-23 is more prominent in the mucosal tissues, such as the intestine, than in the systemic immune compartment enhancing its attractiveness as a therapeutic target in IBD (Uhlig et al., 2006b). A functional receptor for IL-23 (IL-12Rβ1 and IL-23R) (Parham et al., 2002) is expressed on αβ and γδ T cells as well as innate leukocytes (Awasthi et al., 2009; Parham et al., 2002) and IL-23 signaling is mediated predominantly by the signal transducer and activator of transcription 3 (STAT3) (Parham et al., 2002).

The functional activity of IL-23 has been primarily linked to the T helper 17 (Th17) cell subset (McGeachy and Cua, 2008; O'Shea and Murray, 2008). Development of Th17 cells is mediated by a combination of the cytokines TGF-β and IL-6, IL-21, or IL-1, through the molecular activity of the transcription factors RORγt, RORα, IRF-4, AHR, and STAT3 (Dong, 2008; Ahern et al., 2008). Th17 cells are enriched for expression of *Il23r* (Langrish et al., 2005) and IL-23 plays an important role in the sustenance of Th17 cell responses in vivo (Mangan et al., 2006; McGeachy et al., 2009). However, the cellular and molecular pathways through which IL-23 promotes inflammatory responses in vivo are poorly characterized.

Human IBD is associated with increased expression of IL-23 and Th17 cell signature cytokines such as IL-17A and IL-17F (Ahern et al., 2008). Furthermore, genome-wide association studies have identified single-nucleotide polymorphisms (SNPs) in *IL23R* and the *STAT3* loci as CD susceptibility regions (Burton et al., 2007; Duerr et al., 2006). Interestingly, *IL23R* variants are risk factors for both CD and UC and thus contribute to both types of IBD (Duerr et al., 2006). Together, these data highlight *IL23R* as a key player in the pathogenesis of IBD.

Indeed, neutralization of IL-23 has been shown to ameliorate and cure colitis in a number of mouse models of IBD, including colitis induced by naive T cell transfer (Elson et al., 2007; Hue et al., 2006) in which the role for IL-23 has been linked to control of Th17 cell responses (Leppkes et al., 2009). Early studies also revealed a key role for Th1 cell responses in T cell-mediated colitis as both T-bet deficiency in T cells (Neurath et al., 2002) and blockade of IFN-γ-inhibited colitis (Powrie et al., 1994). Genetic ablation of *Il23a* rather surprisingly revealed that IL-23 rather than IL-12 drives the Th1-IFN-γ inflammatory axis in the intestine (Hue et al., 2006), although the mechanisms by which IL-23 promotes Th1 cell responses in vivo is not known.

Pathogenic effector T cell responses in the intestine are normally prevented by the presence of regulatory (Treg) T cells derived from both thymic and peripherally induced Foxp3^+^ Treg (iTreg) cells (Izcue et al., 2009). It has been demonstrated that IL-23 drives intestinal inflammation in part through the inhibition of iTreg cell development in the intestine (Izcue et al., 2008), but precisely how IL-23 controls this process is still poorly understood. IL-23 is also known to drive intestinal inflammation in *Rag1*^−/−^ mice infected with *Helicobacter hepaticus* in the absence of T or B cells (Buonocore et al., 2010; Hue et al., 2006), and this has recently been linked to an IL-23-responsive innate lymphoid population (Buonocore et al., 2010). Thus, IL-23 can promote intestinal pathogenesis via direct stimulation of the innate immune system raising the possibility that the effects on T cell responses are indirect through the activity of IL-23 on innate immune cells.

Here, we have utilized *Il23r^−/−^* mice to assess the role of IL-23R signaling in T cells in the development of chronic colitis. Our results showed that IL-23 drives intestinal but not systemic inflammation through direct effects on T cells. IL-23 signals into T cells promoted their proliferation and accumulation in the colon and favored the emergence of an IL-17A^+^IFN-γ^+^ population of T cells while inhibiting Foxp3 expression. These results indicate that through its actions on T cells, IL-23 modulates both inflammatory and regulatory arms of Th cell responses to orchestrate intestinal inflammation.

## Results

### IL-23R Expression on T Cells Is Required for Intestinal but Not Systemic Inflammation

**Figure 1:**
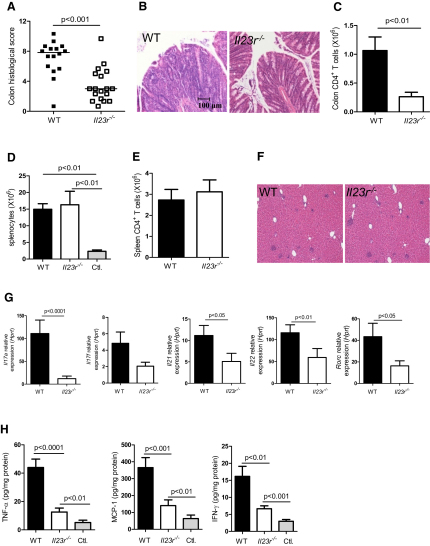
IL-23R Expression on T Cells Is Required for Intestinal but Not Systemic Inflammation C57BL.6.*Rag1^−/−^* mice were transferred with 4 × 10^5^ CD4^+^CD45RB^hi^ T cells from WT or *Il23r^−/−^* donors. Mice were sacrificed when recipients of WT T cells developed clinical signs of disease (∼9 weeks after transfer) and assessed for intestinal and systemic inflammation. (A) Colitis scores. (B) Representative photomicrographs of mid colon sections. (C) Total CD4^+^ T cells in colon. (D) Total splenocytes. (E) Total CD4^+^ T cells in spleen. (F) Representative photomicrographs of liver sections. (G) Expression of cytokine mRNA in colon tissue homogenates, normalized to *Hprt*. (H) Concentration of cytokines in colon tissue homogenates, normalized to total protein. Data represent pooled results from two to three independent experiments. Bars represent the mean, error bars represent the SEM, and each symbol represents an individual mouse, n = 11–16 (WT T cells), n = 13–18 (*Il23r^−/−^* T cells), n = 3–13 (Controls; Ctl.). Statistical significance was determined with the Mann-Whitney test.

To assess the role of IL-23R expression on T cells in the development of colitis, we transferred CD4^+^CD45RB^hi^ cells isolated from wild-type (WT) or *Il23r^−/−^* mice into B and T cell-deficient *Rag1^−/−^* mice, with the latter allowing us to restrict IL-23 responsiveness to the innate immune compartment. As previously described (Powrie et al., 1993; Read et al., 2000), WT CD4^+^CD45RB^hi^ T cells vigorously expand upon transfer and induce both a systemic inflammatory response and severe colitis (Figures 1A–1F). By contrast, the majority of mice restored with *Il23r^−/−^* CD4^+^ T cells developed only minimal intestinal inflammation with little cellular infiltrate or epithelial hyperplasia (Figures 1A and 1B). Transfer of *Il23r^−/−^* T cells led to an 80% reduction in the number of CD4^+^ T cells in the colon compared to WT controls illustrating an important requirement for IL-23R signaling in T cells for their ability to accumulate and orchestrate an inflammatory response in the intestine (Figure 1C).

Despite the impaired mucosal inflammation, transfer of *Il23r^−/−^* T cells led to a potent systemic inflammatory response. Indeed, compared with recipients of IL-23R-sufficient T cells, we observed no difference in splenomegaly (Figure 1D), splenic T cell accumulation (Figure 1E), hepatic infiltrates (Figure 1F), serum concentrations of proinflammatory cytokines and chemokines, such as IL-6, MCP-1, TNF-α, and IFN-γ (Figure S1A available online), or weight loss (Figure S1B) in recipients of *Il23r^−/−^* T cells. Serum IL-22 concentrations were significantly reduced in the absence of IL-23R expression on T cells, indicating a role for IL-23 in the systemic production of certain Th17 cell-associated cytokines. By contrast, the attenuated inflammation observed in the intestine of recipients of *Il23r^−/−^* T cells was associated with reduced expression of a number of Th17 cell signature cytokines including *Il17a*, *Il21*, and *Il22*, as well as in the Th17 cell master transcription factor *Rorc* (Figure 1G). However, unlike the systemic immune compartment, the intestines of recipients of *Il23r^−/−^* T cells had reduced concentrations of more general inflammatory cytokines such as TNF-α, MCP-1, and IFN-γ (Figure 1H).

Together, these data suggest a tissue-specific role for IL-23R signaling in T cells for intestinal inflammation, whereas the systemic inflammatory response is largely independent of the direct effects of IL-23 on T cells.

### IL-23 Regulates Intestinal Th Cell Responses through Direct Effects on T Cells

**Figure 2:**
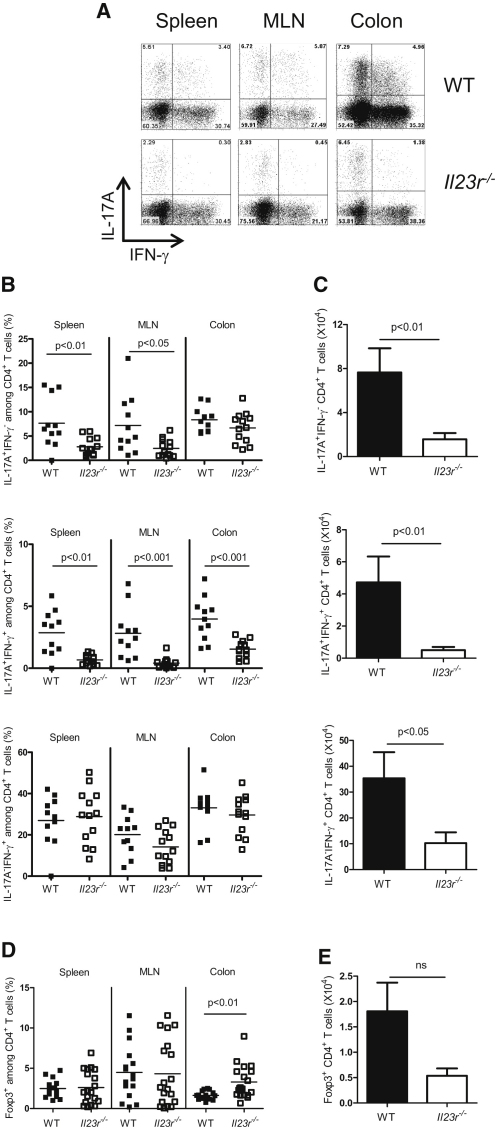
IL-23 Regulates Intestinal Th Cell Responses via Direct Effects on T Cells C57BL.6.*Rag1^−/−^* mice were transferred with 4 × 10^5^ CD4^+^CD45RB^hi^ T cells from WT or *Il23r^−/−^* donors and sacrificed when recipients of WT T cells developed clinical signs of disease (∼9 weeks after transfer). IL-17A, IFN-γ, and Foxp3 amounts in T cells from various tissues were assessed by intracellular flow cytometry after in vitro restimulation with PMA and ionomycin. (A) Representative flow cytometry plots of CD4^+^ T cells from the spleen, MLN, and colon. Numbers in quadrants represent frequencies. (B) Frequencies of IL-17A^+^ and/or IFN-γ^+^ T cells in the spleen, MLN, and colon. (C) Total numbers of IL-17A^+^ and/or IFN-γ^+^ CD4^+^ T cells in the colon. (D) Frequencies of Foxp3^+^ CD4^+^ T cells in the spleen, MLN, and colon. (E) Total number of Foxp3^+^ CD4^+^ T cells in the colon. Data represent pooled results from two to three independent experiments, bars represent the mean, error bars represent the SEM, and each symbol represents an individual mouse. Statistical significance was determined with the Mann-Whitney test, n = 11–16 (WT), n = 13–18 (*Il23r^−/−^*).

T cell transfer colitis is associated with the accumulation of Th1 and Th17 cells in the intestine (Hue et al., 2006; Izcue et al., 2008). To analyze the direct effects of IL-23R signals on Th cell subset differentiation in the intestine, we isolated cells from the spleen, mesenteric lymph node (MLN), and colon of mice transferred with WT or *Il23r^−/−^* CD4^+^ T cells and performed intracellular flow cytometric analysis of IL-17A and IFN-γ expression. Three distinct populations emerged in *Rag1^−/−^* recipients of WT CD4^+^ T cells: IL-17A^+^IFN-γ^−^, IL-17A^−^IFN-γ^+^, and IL-17A^+^IFN-γ^+^ (Figure 2A). We found significant reductions in the IL-17A^+^IFN-γ^−^ population of T cells in the spleen and MLN but not in the intestine of recipients of *Il23r^−/−^* T cells (Figure 2B). However, the IL-17A^+^IFN-γ^+^ T cell population was significantly reduced in all compartments, including the intestine, in recipients of *Il23r^−/−^* T cells, indicating a role for IL-23 in promoting this effector cell phenotype (Figure 2B). By contrast, there was no difference in the frequency of IL-17A^−^IFN-γ^+^ T cells in any compartment of *Rag1^−/−^* mice that received WT or *Il23r^−/−^* T cells (Figure 2B), demonstrating that *Il23r^−/−^* T cells were not defective in their ability to differentiate into IFN-γ producing Th1 cells. However, there was a reduced number of all three effector populations, including IL-17A^−^IFN-γ^+^ T cells in the intestine of mice transferred with *Il23r^−/−^* T cells (Figure 2C). Thus, although IL-23 was not required for Th1 cell development, the expansion and/or accumulation of this population was facilitated by an IL-23-driven T cell response.

Previously, we have shown that IL-23 promotes intestinal inflammation in part through its ability to antagonize iTreg cell development in the intestine (Izcue et al., 2008). Whether this is a direct or indirect activity of IL-23 on T cells was not established. We observed that *Il23r^−/−^* T cells gave rise to higher frequencies of Foxp3^+^ T cells in the intestine when transferred into *Rag1^−/−^* recipients as compared to their WT counterparts (Figure 2D), although total numbers were not significantly different (Figure 2E). This suggests that IL-23-driven Foxp3 inhibition can be mediated by the direct activity of IL-23 on T cells.

### IL-23 Controls T Cell Accumulation in the Intestine through Effects on Proliferation

**Figure 3:**
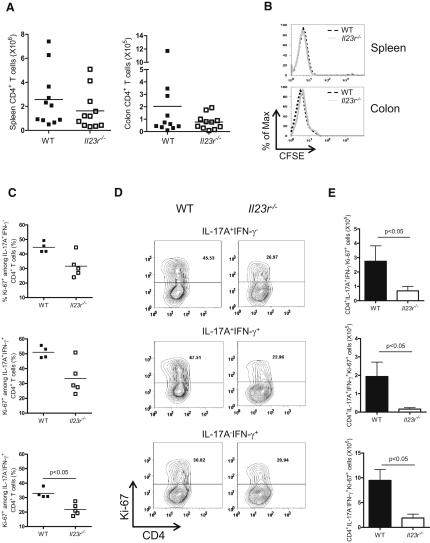
IL-23 Directly Promotes Intestinal T cell Proliferation (A and B) C57BL.6.*Rag1^−/−^* mice were transferred with 4 × 10^6^ CFSE-labeled CD4^+^CD45RB^hi^ T cells from WT or *Il23r^−/−^* donors and sacrificed 12 days after transfer. (A) Total CD4^+^ T cell numbers and (B) representative flow cytometry plots showing CFSE dilution profiles of T cells from the spleen and colon are presented. (C–E) C57BL.6.*Rag1^−/−^* mice were transferred with 4 × 10^5^ CD4^+^CD45RB^hi^ T cells from WT or *Il23r^−/−^* donors and sacrificed when recipients of WT T cells developed clinical signs of disease (∼6 weeks after transfer). IL-17A, IFN-γ, and Ki67 expression in CD4^+^ T cells from the colon were assessed by intracellular flow cytometry after in vitro restimulation with PMA and ionomycin. (C) Frequencies, (D) representative flow cytometric plots (numbers in gate represent frequencies), and (E) total numbers of IL-17A^+^IFN-γ^−^Ki-67^+^, IL-17A^+^IFN-γ^+^Ki-67^+^, and IL-17A^−^IFN-γ^+^Ki-67^+^ CD4^+^ T cells in the colon. Data represent pooled results from two independent experiments (A and B) or of a single experiment (C–E), bars represent the mean, error bars represent the SEM, and each symbol represents an individual mouse. Statistical significance was determined with the Mann-Whitney test, n = 4-11 (WT), n = 5-11 (*Il23r^−/−^*).

The failure of *Il23r^−/−^* T cells to accumulate in the inflamed colon could reflect a homing defect or a failure to proliferate and/or survive in the colon. To assess the ability of *Il23r^−/−^* T cells to home to the colon, we transferred *Rag1^−/−^* mice with CFSE-labeled WT or *Il23r^−/−^* CD4^+^ T cells and sacrificed recipients 12 days after transfer, prior to the onset of inflammation. We found equal numbers of T cells in the spleen and colon at this time point, indicating that *Il23r^−/−^* CD4^+^ T cells were not defective in their ability to migrate to the intestine (Figure 3A). In addition, *Il23r^−/−^* CD4^+^ T cells are not impaired in their ability to express the chemokine receptors *Ccr6*, *Cxcr3*, *Ccr2*, and *Ccr5* (Figure S2A). Furthermore, both populations of T cells had diluted CFSE to the same degree (Figure 3B), demonstrating that *Il23r^−/−^* CD4^+^ T cells did not display an early impairment in division once present in the colon. Next, we assessed the proliferative capacity of WT and *Il23r^−/−^* T cells later in the response. Thus, *Rag1^−/−^* mice were transferred with WT or *Il23r^−/−^* CD4^+^ T cells and sacrificed upon clinical signs of inflammation in WT transferred recipients. Proliferation was assessed by measuring the frequency of Ki-67^+^ CD4^+^ T cells with intracellular flow cytometry. Under these conditions, we found reduced frequencies of Ki-67^+^ cells among all three effector Th cell populations in the colon of *Il23r^−/−^* T cell-transferred *Rag1^−/−^* recipients compared to those recovered from WT T cell-transferred recipients (Figures 3C and 3D). In *Il23r^−/−^* T cell-transferred mice, there were also marked reductions in the total number of proliferating effector Th populations (IL-17A^+^IFN-γ^−^, IL-17A^−^IFN-γ^+^, and IL-17A^+^IFN-γ^+^) in the colon (Figure 3E). By contrast, equivalent frequencies of Ki-67^+^ T cells were observed in WT and *IL23r^−/−^* effector T cell populations recovered from the spleen (Figure S2B and S2C). Together, these data indicate that IL-23 is an important proliferative signal for colonic T cells, and such a finding may directly account for the impaired ability of *Il23r^−/−^* T cells to accumulate in the intestine.

### IL-23 Controls T Cell Accumulation in the Intestine through a Cell-Extrinsic Mechanism

**Figure 4:**
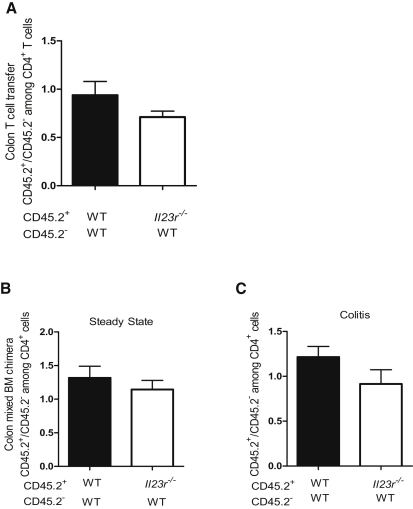
IL-23 Controls T cell Accumulation in the Intestine via a Cell-Extrinsic Mechanism C57BL.6.*Rag1^−/−^* mice were transferred with 1:1 mixtures of CD45.2^−^ (WT) + CD45.2^+^ (WT) or CD45.2^−^ (WT) + CD45.2^+^(*Il23r^−/−^*) CD4^+^CD45RB^hi^ T cells. Mice were sacrificed upon development of clinical signs of inflammation (∼8 weeks) and populations of T cells were identified on the basis of the expression of CD45.2. (A) Ratio of CD45.2^+^/CD45.2^−^ CD4^+^ T cells in colon. Data represent pooled results from two independent experiments; n = 15 (WT + WT), n = 12 (WT + *Il23r^−/−^*). Bars represent the mean ± SEM. (B and C) Sublethally irradiated C57BL.6.*Rag1^−/−^* mice were reconstituted with 1:1 mixtures of CD45.2^−^ (WT) + CD45.2^+^ (WT) or CD45.2^−^ (WT) + CD45.2^+^(*Il23r^−/−^*) bone marrow cells. Ratio of CD45.2^+^/CD45.2^−^ T cells recovered from the colon during steady state (B) or during colitis induced by infection with *Helicobacter hepaticus* plus treatment with a blocking IL-10R mAb (C). Data represent results from a single experiment; n = 5–7 (WT + WT), n = 5–7 (WT + *Il23r^−/−^* T cells). Bars represent the mean ± SEM.

Next, we assessed the ability of *Il23r^−/−^* T cells to compete with WT T cells under inflammatory conditions. Using a cotransfer system (Figure S3A), we administered an equal number of congenic CD45.2^−^ WT and CD45.2^+^ *Il23r^−/−^* naive CD4^+^ T cells into *Rag1^−/−^* recipients. For a control, we also transferred a 1:1 ratio of CD45.2^−^ and CD45.2^+^ WT naive CD4^+^ T cells and allowed mice to develop wasting disease and colitis. Assessment of the relative contribution of CD4^+^ cells in the MLN and colon that derived from the transferred CD45.2^−^ or CD45.2^+^ T cell populations revealed that IL-23R-deficient CD4^+^ T cells were indistinguishable from WT CD4^+^ T cells in their ability to accumulate in the MLN and inflamed colon of colitic mice (Figure S4 and Figure 4A). These results indicate that the presence of IL-23R-sufficient T cells and their subsequent downstream inflammatory signals overcomes the requirement for cell-intrinsic IL-23R signaling for T cell accumulation in the intestine.

Although the T cell transfer model offers a unique opportunity to study the contributions of various cell types to intestinal immune pathology, the increased T cell proliferation after transfer to lymphopenic *Rag1^−/−^* hosts may overcome a cell intrinsic role for IL-23R in intestinal accumulation of T cells. To test this, we generated mixed bone marrow chimeras by using a 1:1 ratio of CD45.2^−^ WT bone marrow with either CD45.2^+^ WT or CD45.2^+^ *Il23r^−/−^* bone marrow (Figure S3B) to assess the capacity of *Il23r^−/−^* T cells to compete for accumulation in the colon in a lymphocyte-replete host. As shown (Figure 4B), even under homeostatic conditions, in the presence of WT T cells, *Il23r^−/−^* T cells showed no deficiency in their capacity to accumulate in the colon and to compete with WT T cells for this niche. To test whether this was also the case during intestinal inflammation, we infected similarly generated chimeras with the intestinal pathogen *Helicobacter hepaticus* and treated them with a blocking IL-10R mAb, which we have previously shown leads to IL-23-dependent colitis and typhlitis (Kullberg et al., 2006). In these mixed chimeras, under inflammatory conditions, we found a similar contribution to the CD4^+^ T cell pool in the colon from WT and *Il23r^−/−^* T cells (Figure 4C). These results confirm our observations in the T cell transfer model and show that the presence of IL-23R^+^ T cells is sufficient to drive the accumulation of *Il23r^−/−^* T cells in the gut by a cell-extrinsic mechanism. Thus, *Il23r^−/−^* T cells are not intrinsically defective in their ability to compete and accumulate under inflammatory conditions, but require an IL-23-driven T cell response to do so. Therefore, although IL-23R signals in T cells are required for their effective accumulation, the effects are not cell autonomous.

### IL-23 Regulates the Balance between Effector and Regulatory Th Cells through Cell-Intrinsic Mechanisms

**Figure 5:**
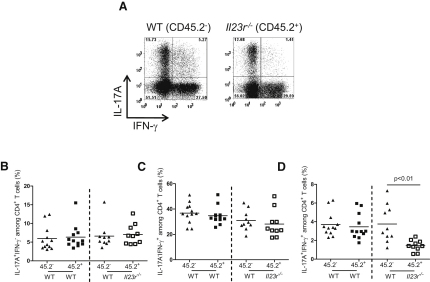
IL-23 Modulates Th17 Cell Phenotype via a Direct Cell-Intrinsic Mechanism C57BL.6.*Rag1^−/−^* mice were transferred with 1:1 mixtures of CD45.2^−^ (WT) + CD45.2^+^ (WT), or CD45.2^−^ (WT) + CD45.2^+^(*Il23r^−/−^*) CD4^+^CD45RB^hi^ T cells. Mice were sacrificed upon development of clinical signs of inflammation (∼8 weeks) and IL-17A and IFN-γ levels in colonic CD4^+^ T cells were assessed by intracellular flow cytometry after in vitro restimulation with PMA and ionomycin. (A) Representative flow cytometry plots of CD4^+^ T cells isolated from the colon; numbers in quadrants represent frequencies. (B–D) Frequencies of IL-17A^+^ and/or IFN-γ^+^ CD4^+^ T cells in the colon of cotransferred mice. Data represent pooled results from two independent experiments. Bars represent the mean; each symbol represents an individual mouse. Statistical significance was determined with the Mann-Whitney test, n = 12 (WT + WT), n = 10 (WT + *Il23r^−/−^*).

Our transfers of *Il23r^−/−^* T cells into *Rag1^−/−^* mice revealed a role for IL-23R signaling in T cells in influencing the balance of Th17 and Foxp3^+^ Treg cells in the intestine. However, we could not rule out the possibility that the decrease in IL-17A^+^IFN-γ^+^ T cells and the increase in Foxp3^+^ Treg cell frequency we observed after transfer of *Il23r^−/−^* T cells was secondary to the lack of inflammation in this setting. To circumvent this, we utilized the cotransfer system described above in which cotransfer of WT and *Il23r^−/−^* T cells led to intestinal inflammation and similar accumulation of IL-23R-sufficient and IL-23R-deficient T cells. Consistent with our observations in single-transfer experiments (Figure 2B), in cotransfer experiments we observed equivalent differentiation of IL-17A^+^IFN-γ^−^ and IL-17A^−^IFN-γ^+^ cells among the WT and *Il23r^−/−^* populations of T cells in the colon (Figures 5A–5C). By contrast, the frequency of IL-17A^+^IFN-γ^+^ cells was significantly reduced among the *Il23r^−/−^* pool of CD4^+^ T cells in the intestine (Figures 5A and 5D), demonstrating a cell-intrinsic role for IL-23 in the in vivo modulation of the Th17 effector cell phenotype. Thus, even under inflammatory conditions in which *Il23r^−/−^* T cells accumulate normally in the intestine, their differentiation into IL-17A^+^IFN-γ^+^ CD4^+^ T cells is impaired, indicating that this is directly controlled by cell-intrinsic IL-23R signals.

**Figure 6:**
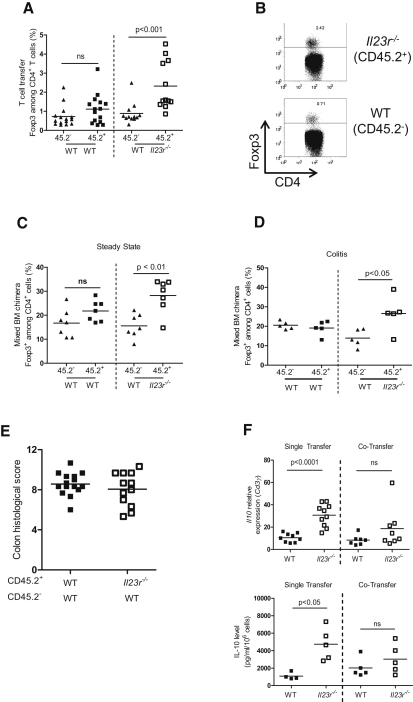
IL-23 Inhibits Intestinal iTreg Cell Generation via a Direct Cell-Intrinsic Mechanism (A and B) C57BL.6.*Rag1^−/−^* mice were transferred with 1:1 mixtures of CD45.2^−^ (WT) + CD45.2^+^ (WT) or CD45.2^−^ (WT) + CD45.2^+^(*Il23r^−/−^*) CD4^+^CD45RB^hi^ T cells. Mice were sacrificed upon development of clinical signs of inflammation (∼8 weeks). (A) The frequency of Foxp3^+^ cells among T cells in the colon was assessed by intracellular flow cytometry. (B) Representative FACS plots showing Foxp3 expression in CD4^+^ T cells in the colon; numbers in quadrants represent frequencies. Data represent pooled results from two independent experiments, n = 15 (WT + WT), n = 12 (WT + *Il23r^−/−^*). (C and D) Sublethally irradiated C57BL.6.*Rag1^−/−^* mice were reconstituted with 1:1 mixtures of CD45.2^−^ (WT) + CD45.2^+^ (WT) or CD45.2^−^ (WT) + CD45.2^+^(*Il23r^−/−^*) bone marrow cells. The frequency of Foxp3^+^ cells among CD4^+^ cells in the colon was assessed by intracellular FACS during steady state (C) or during colitis induced by infection with *Helicobacter hepaticus* plus treatment with a blocking IL-10R mAb (D). Data represent results from a single experiment; n = 5–7 (WT + WT), n = 5–7 (WT + *Il23r^−/−^* T cells). (E) Colitis scores from mice in (A); data represent pooled results from two independent experiments. (F) *Il10* mRNA levels and protein levels in supernatants after restimulation of CD4^+^ T cells purified from the colons of C57BL.6.*Rag1^−/−^* mice transferred with WT or *Il23r^−/−^* CD4^+^CD45RB^hi^ T cells as described in Figure 1 (single transfer) or from recipients of 1:1 mixtures of WT + *Il23r^−/−^* CD4^+^CD45RB^hi^ T cells as described above (cotransfer). Data represent pooled results from two independent experiments (top, n = 7–10 per group) or from a single experiment (bottom, n = 5 per group). Bars represent the mean; each symbol represents an individual mouse; statistical significance was determined with the Mann-Whitney test.

Analysis of Foxp3 expression in these cotransfer experiments also revealed a cell-intrinsic role for IL-23R signaling in T cells in the inhibition of Foxp3 expression. As shown (Figures 6A and 6B), in the inflamed colon there was a 2- to 3-fold increase in the frequency of Foxp3^+^ cells among *Il23r^−/−^* T cells compared to cotransferred WT T cells. Given that only a very low proportion of naive T cells differentiated into Foxp3^+^ iTreg cells after transfer into *Rag1^−/−^* recipients, we sought to confirm these findings in lymphocyte-replete hosts in the presence or absence of intestinal inflammation. We performed similar analysis of Foxp3 expression in the mixed bone marrow chimeras described above, both under steady state conditions and in circumstances where intestinal inflammation was induced by infection with *H. hepaticus* with concomitant administration of a blocking IL-10R mAb. We again observed a significant increase in the frequency of Foxp3^+^ cells derived from *Il23r^−/−^* T cells compared to WT T cells under both homeostatic (Figure 6C) and inflammatory conditions (Figure 6D). Together, these data indicate that IL-23R signaling in T cells is a tissue-specific modulator of T cell function in the intestine where it negatively regulates Foxp3 expression in a cell-intrinsic fashion.

Despite the increased frequencies of Treg cells observed in the colon of mice transferred with *Il23r^−/−^* T cells, the incidence and severity of colitis in mice receiving a mixture of CD45.2^−^ WT plus CD45.2^+^ WT or CD45.2^−^ WT plus CD45.2^+^ *Il23r^−/−^* T cells was almost identical, indicating that *Il23r^−/−^* T cells do not possess dominant suppressive activity (Figure 6E). We therefore assessed whether the regulatory properties of *Il23r^−/−^* T cells, specifically IL-10 production, were altered in the cotransfer system compared to single transfers. Consistent with reduced intestinal inflammation (Figures 1A and 1B), colonic *Il23r^−/−^* T cells from single-transfer experiments expressed significantly greater amounts of *Il10* mRNA and secreted higher amounts of IL-10 protein than WT T cells (Figure 6F). By contrast, *Il23r^−/−^* T cells isolated from the colon of mice cotransferred with WT cells did not exhibit this enhanced IL-10 expression and secretion (Figure 6F). Thus, in the presence of IL-23R-responsive WT T cells there is a reduction in the ability of *Il23r^−/−^* T cells to produce IL-10, which may explain the development of colitis in mice given a mixture of WT and *Il23r^−/−^* T cells, despite increases in the percentage of Foxp3^+^ Treg cells.

## Discussion

Immune responses in the intestine require a delicate balance between effector and regulatory pathways and perturbation of this network can result in chronic intestinal inflammation. IL-23 has been shown to play a key role in both innate and T cell mediated chronic colitis in mouse models (Hue et al., 2006; Kullberg et al., 2006; Uhlig et al., 2006b; Yen et al., 2006) and *IL23R* has been identified as a risk gene in IBDs (Burton et al., 2007; Duerr et al., 2006). However, precisely which cell types it works on to drive the intestinal inflammatory response was not known. Here, utilizing IL-23R-deficient CD4^+^ T cells, we demonstrated that intestinal, but not systemic, inflammation required the direct stimulation of CD4^+^ T cells by IL-23 and could not be mediated by IL-23-driven innate immune or IL-23-independent T cell responses alone. We further showed that IL-23 signaling in T cells played a crucial role in their proliferation and accumulation in the colon and promoted the emergence of IL-17A^+^IFN-γ^+^ double-producing CD4^+^ T cells while inhibiting the induction of Foxp3^+^ Treg cells in the gut. These data suggest that the tissue-specific role of IL-23 in orchestrating intestinal inflammation is mediated through direct effects on T cells that promote their accumulation and pathogenic effector function in the intestine.

The inability of IL-23R-deficient T cells to accumulate in the colon upon transfer into *Rag1^−/−^* hosts, but their unimpaired ability to mount inflammatory responses in the spleen and liver, suggests that IL-23 signaling in T cells is not required for general T cell reconstitution of lymphopenic mice but is specifically required for the accumulation of effector T cells in the intestine. Mechanistic analysis showed this was not due to impaired migration but rather a reduction in the proliferation of effector T cells locally in the colon. This proliferative defect was observed among colonic Th17 and Th1 cells, explaining the reduction in both Th1 and Th17 cell numbers in the colon in the absence of IL-23R signaling in T cells. Recently, we have found that STAT3-deficient T cells fail to mount either systemic or intestinal inflammation in T cell transfer colitis (Durant et al., 2010). These results suggest that IL-23 is a major mediator of STAT3 signaling in T cells in the intestinal but not systemic immune compartment. Our results also raise the possibility that the requirement for RORγt expression in T cells for development of T cell transfer colitis (Leppkes et al., 2009) reflects the known activities of RORγt in promoting *Il23r* expression (Zhou et al., 2007) on T cells.

In stark contrast to the inability of adoptively transferred *Il23r^−/−^* T cells to accumulate in the intestine, the presence of WT T cells, in either the T cell cotransfer experiments or in the mixed bone marrow chimeras, facilitated the efficient accumulation of *Il23r^−/−^* T cells in the colon. This suggests that an IL-23R-sufficient T cell is required to initiate the inflammatory cascade and produces factors in response to IL-23 that sustain the proliferation and accumulation of IL-23R-deficient T cells. Such signals may be of paramount importance in the context of intestinal inflammation, for which IL-23-driven pathology is characterized by the accumulation of both Th1 and Th17 cells in the intestine (Hue et al., 2006; Izcue et al., 2008). These findings contrast with those described in models of CNS inflammation, where Th1 and Th17 cell responses are thought to be antagonistic. Furthermore, a cell-intrinsic role for IL-23R expression in Th17 cell accumulation in the brain during EAE was recently described, which could not be overcome by the presence of WT T cells (McGeachy et al., 2009). Taken together, these data indicate that the requirements for direct IL-23 signals in T cell accumulation at inflammatory sites are likely to be context dependent, modulated by the tissue environment and the presence of other inflammatory cells.

In addition to impaired accumulation in the intestine, *Il23r^−/−^* T cells gave rise to significantly reduced frequencies of T cells secreting IL-17A, but not IFN-γ, compared to WT T cells. This suggests a specific role for IL-23R signaling in T cells for the differentiation, proliferation, or survival of Th17 cells. Similar results have been observed in EAE models where IL-23 was found to drive increased accumulation of Th17 cells (Awasthi et al., 2009; McGeachy et al., 2009). Recent studies suggest that Th17 cells exhibit flexibility of function and can modify their phenotype, including acquisition of IFN-γ production in response to IL-12 or IL-23 in vitro or during induction of diabetes and colitis (Bending et al., 2009; Lee et al., 2009). Notably, IL-17A^+^IFN-γ^+^ CD4^+^ T cells are prominent in T cell-dependent colitis models (Hue et al., 2006; Kullberg et al., 2006) and have also been recovered from the intestinal lesions of human CD patients (Annunziato et al., 2007; Cosmi et al., 2008). Our results extend these studies to show that in the intestine the emergence of IL-17A^+^IFN-γ^+^ double-producing Th cells, but not IL-17A^+^IFN-γ^−^ Th cells, requires T cell-intrinsic IL-23 signaling. Together, these results suggest that IL-23 is an important modifier of the Th17 cell phenotype in the intestine, promoting the emergence of IL-17A^+^IFN-γ^+^ Th cells. The dependence of these cells on IL-23 suggests they play an important role in pathogenesis. The ability of IL-23 to promote IFN-γ expression in Th17 cells is T-bet dependent in vitro (Lee et al., 2009), raising the possibility that in addition to driving conventional Th1 cell responses, the requirement for T-bet in T cell transfer colitis (Neurath et al., 2002) involves IL-23-dependent modulation of the Th17 cell phenotype. Indeed, there is evidence of a role for T-bet in the pathogenicity of Th17 cells in EAE (Yang et al., 2009).

Under homeostatic conditions, the intestine has been shown to be a preferential site for the differentiation of Foxp3^+^ iTreg cells (Coombes et al., 2005). Our previous work showed that during intestinal inflammation, IL-23 could antagonize development of Foxp3^+^ Treg cells, facilitating the development of intestinal inflammation (Izcue et al., 2008). Whether IL-23 works directly on T cells or indirectly through APC to mediate this effect was not established. Here, we show that IL-23 inhibits the accumulation of Foxp3^+^ Treg cells in the intestine through a direct cell intrinsic mechanism. This effect was observed in T cell transfer colitis as well as in lymphocyte-replete bone marrow chimeras in the presence or absence of intestinal inflammation, indicating that the effects of IL-23R signaling into T cells on Foxp3 expression are not secondary to a lack of inflammation or the lymphopenic *Rag1^−/−^* environment. Recently it has been shown that, prior to differentiating into Th17 or Foxp3^+^ iTreg, T cells may pass through a RORγt^+^Foxp3^+^ intermediate stage (Lochner et al., 2008; Zhou et al., 2008). Such RORγt^+^Foxp3^+^ double positive cells are more frequent in the intestine raising the possibility that IL-23 acts directly on these cells to promote Th17 differentiation and inhibit induction of Foxp3^+^ Treg cells. Although IL-23R deficiency was associated with impaired EAE, there was no increase in the frequency of Foxp3^+^ Treg cells in the CNS among *Il23r^−/−^* T cells in mixed bone-marrow chimaeras with active EAE (McGeachy et al., 2009). Differences in the actions of IL-23 on Treg cells in the gut and CNS may reflect the additional influence of the microbiota which is known to influence accumulation of Treg in the intestine (Ivanov et al., 2008). Alternatively, other STAT3 signaling cytokines such as IL-6 and IL-21 may be more important negative regulators of Foxp3 expression outside the intestine (Bettelli et al., 2006; Korn et al., 2007; Nurieva et al., 2007; Zhou et al., 2007). In contrast with the induction of Foxp3^+^ Treg cells in the periphery, there is evidence that in the tumor microenvironment, STAT3 signaling through IL-23R expression increases Foxp3 and IL-10 expression by Treg cells (Kortylewski et al., 2009). These opposing roles of IL-23R signaling on Foxp3 expression in the tumor and gut may reflect differences in the tissue microenvironments or the differentiation state of responding T cells. One possibility that we favor is that in the intestine the effects of IL-23 are to restrain iTreg cell development, whereas in the tumor microenvironment IL-23 may act on established thymic-derived Treg cells to enhance their function.

Reduced colitogenic activity among *Il23r^−/−^* T cells was also associated with increased expression of IL-10. These results are consistent with our earlier finding that IL-10 plays a functional role in the amelioration of intestinal inflammation in *Il23a^−/−^* mice (Izcue et al., 2008). By contrast with the cell-intrinsic role of IL-23R in the negative regulation of Foxp3 expression, we found that the presence of WT cells was sufficient to abrogate increased IL-10 expression by *Il23r^−/−^* T cells. The ability of IL-23 to act through a cell-extrinsic mechanism to reduce T cell-derived IL-10 may be a further pathway through which IL-23 promotes intestinal inflammation. Further studies are required to identify the molecular mechanism involved.

In summary, we describe a crucial role for IL-23-driven T cell responses in the development of intestinal but not systemic inflammation. Our findings underscore genetic studies that pinpoint a role for IL-23R-mediated pathways in IBD pathogenesis (Burton et al., 2007; Duerr et al., 2006) and provide insight into the mechanism of action of IL-23 in vivo. Our results highlight the dual nature of IL-23 activity on T cells and suggest that blockade of IL-23 will ameliorate intestinal inflammation by inhibiting the accumulation and pathogenic activity of helper T cells and promote mucosal tolerance by facilitating expansion of iTreg cells and production of IL-10.

## Experimental Procedures

### Mice

Wild-type C57BL.6., C57BL.6.*Il23r^−/−^*, congenic B6.SJL-*Cd45.1*, and C57BL.6.*Rag1^−/−^* mice were bred and maintained under specific pathogen-free conditions in accredited animal facilities at the University of Oxford and Merck Research Laboratories. Experiments were conducted in accordance with the UK Scientific Procedures Act of 1986. Mice were routinely screened for *Helicobacter spp.* and were >6 weeks old when used.

### Generation of Mixed Bone Marrow Chimeras

Bone marrow isolated from C57BL.6.or C57BL.6.6 *Il23r^−/−^* mice was mixed in a 1:1 ratio with bone marrow taken from B6.SJL- *Cd45.1* mice and injected intravenously into gamma-irradiated (5.5 Gy, 550 rad) C57BL.6.*Rag1^−/−^* recipients and chimaeras were used in experiments >20 weeks after injection.

### Transfer of Naive CD4^+^CD45RB^hi^ T Cells

Naive CD4^+^CD45RB^hi^ T cells were purified (>98%) from spleens of C57BL.6 or C57BL.6 *Il23r^−/−^* mice via FACS sorting as previously described (Izcue et al., 2008) with a cell sorter (MoFlo; DakoCytomation). In cotransfer experiments, C57BL.6 or C57BL.6 *Il23r^−/−^* naive CD4^+^CD45RB^hi^ T cells were mixed 1:1 with naive CD4^+^CD45RB^hi^ T cells isolated from congenic B6.SJL-*Cd45.1* mice. Naive T cell suspensions were washed in sterile PBS and sex-matched *Rag1^−/−^* recipient mice received 4 × 10^5^ CD4^+^CD45RB^hi^ T cells by intraperitoneal (i.p.) injection. For cotransfer experiments, 8 × 10^5^ cells were transferred per mouse in one of the two experiments. For mice sacrificed on day 12 after transfer, 4 × 10^6^ CD4^+^CD45RB^hi^ T cells labeled with 5 μM CFSE were transferred per mouse.

### Assessment of Intestinal Inflammation

Mice were monitored regularly and sacrificed when symptoms of clinical disease (significant weight loss and/or diarrhea) became apparent. Intestinal inflammation was assessed as previously described (see “Assessment of Intestinal Inflammation” in the Supplemental Experimental Procedures) (Izcue et al., 2008).

### Isolation of Leukocyte Subpopulations and FACS

Cell suspensions from spleen, MLN, and the lamina propria were prepared as described previously (Uhlig et al., 2006a). Cells were surface stained for flow cytometry with combinations of the following panel of antibodies: anti-CD4 conjugated to PerCp or FITC, anti-mouse TCR-β conjugated to APC, and anti-CD45.2 conjugated to FITC. Intracellular staining was performed as follows: cells were restimulated for 4 hr as previously described (Hue et al., 2006). Cells were washed and incubated with anti-Fc receptor (anti CD16/32 from eBioscience) to prevent nonspecific staining at 4°C. Cells were washed and stained for surface markers indicated above, washed again, and fixed overnight in eBioscience Fix/Perm buffer at 4°C. Cells were then washed and then permeabilized in eBioscience Permeabilisation buffer for 1 hr at 4°C, and subsequently, cells were stained with anti-IL-17A conjugated to PE, anti-IFN-γ conjugated to APC or FITC (both from BD Biosciences), anti-Foxp3 conjugated to APC or FITC (from eBiosciences), and anti-Ki-67 conjugated to FITC (BD Biosciences) or appropriate isotype controls (BD Biosciences) for 30 min at 4°C. Cells were washed twice and acquired with a FACSCalibur, FACSort (BD Biosciences), or Cyan (Dako), and analysis was performed with FlowJo (Tree Star) software.

### Cytokine Quantitation in Intestinal Tissues and Serum

Frozen samples of the proximal, mid, and distal colon were processed as described previously (Hue et al., 2006) with homogenization performed with a FastPrep 24 homogenizer (MP Biomedicals) with lysing matrix D beads (MP Biomedicals). Colonic cytokines were measured with cytometric bead assay (BD Biosciences) (TNF-α, IFN-γ, and MCP-1) and normalized to protein levels as determined by Bradford Assay (Bio-Rad). Alternatively cytokines were quantified by RT-qPCR (Hue et al., 2006). For determination of IL-10 protein levels and gene expression, CD4^+^ T cells were purified from the colon with a cell sorter (MoFlo; DakoCytomation) and cells were restimulated overnight as above without Brefeldin A. Sequences for primers sets and probes are described in Table S1. *Ccr6*, *Ccr5*, *Ccr2*, and *Cxcr3* qPCR primer sets are taken from PrimerBank (Spandidos et al., 2008, 2010; Wang and Seed, 2003). Serum cytokines (IL-17A, IL-22, TNF- α, IFN-γ, MCP-1, and IL-6) and IL-10 levels in supernatants of overnight cultures were assessed with Flow Cytomix (Bender MedSystems) in accordance with the manufacturer's protocol.

### Bacteria

*Helicobacter hepaticus* was grown and used for colitis induction with concomitant administration of a blocking IL-10R mAb as previously described (see “Bacteria” in the Supplemental Experimental Procedures) (Buonocore et al., 2010).

### Statistics

The nonparametric Mann-Whitney test was used for assessment of statistical significance (Olsen, 2003). Differences were considered statistically significant when p < 0.05.

## Supplemental Information

Document S1. Four Figures, One Table, and Supplemental Experimental Procedures
