# Interleukin-23 Drives Intestinal Inflammation through Direct Activity on T Cells

**DOI:** 10.1016/j.immuni.2011.02.018

**Published:** 2011-03-25

**Authors:** Philip P. Ahern, Chris Schiering, Sofia Buonocore, Mandy J. McGeachy, Dan J. Cua, Kevin J. Maloy, Fiona Powrie

(Immunity *33*, 279–288; August 27, 2010)

In this paper, there was an error in Figure 1F. The histology panel on the left is incorrect; it is the same as the image on the right taken in a different frame. Thus, both images depict the liver of a *Rag1*^−/−^ host transferred with *Il23r*^−/−^ T cells. The correct image of a representative liver section of a *Rag1*^−/−^ host transferred with WT T cells is published here, along with the rest of the figure. The authors regret any inconvenience this error has caused.

## Figures and Tables

**Figure 1:**
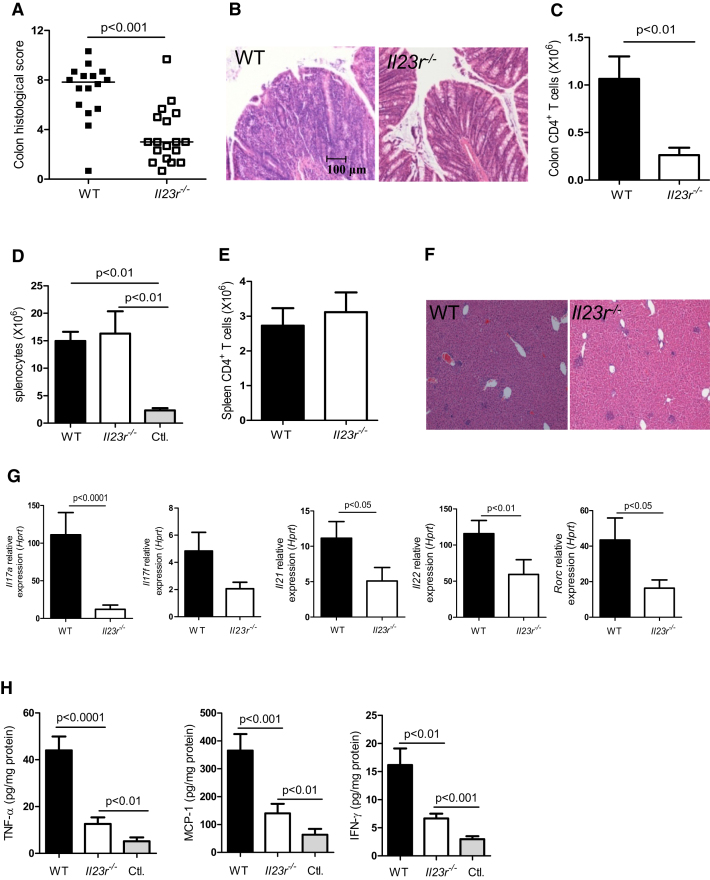
IL-23R Expression on T Cells Is Required for Intestinal but Not Systemic Inflammation

