# Supplementary material for: Interleukin-23 Drives Intestinal Inflammation through Direct Activity on T Cells
Source: Immunity. 2010 Aug 27;33(2-2):279–88. doi: 10.1016/j.immuni.2010.08.010 (PMC3078329; doi:10.1016/j.immuni.2010.08.010)
Supplement: Document S1. Four Figures, One Table, and Supplemental Experimental Procedures [file mmc1.pdf]

Immunity, Volume *33*

Supplemental Information

Interleukin-23 Drives Intestinal Inflammation through Direct Activity on T Cells

Philip P. Ahern, Chris Schiering, Sofia Buonocore, Mandy J. McGeachy, Dan J. Cua, Kevin J. Maloy, and Fiona Powrie

Figure S1

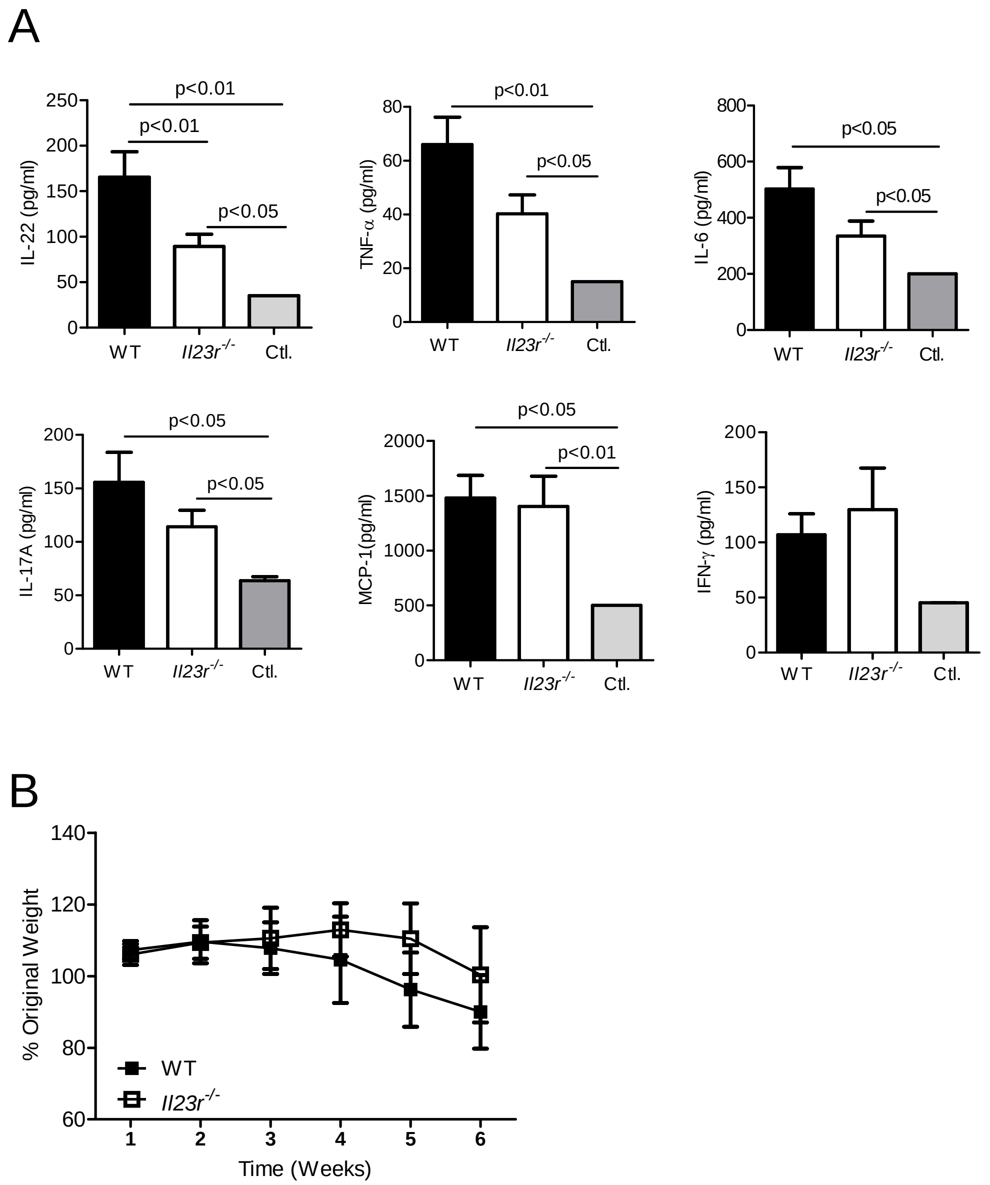

Figure S1. Systemic Inflammation does not Require IL-23R Expression on T cells

C57BL.6.*Rag1*$^{-/-}$ mice were transferred with 4 x 10^5 $CD4^+CD45RB^{hi}$ T cells from WT or *Il23r*$^{-/-}$ donors. Mice were sacrificed when recipients of WT T cells developed clinical signs of disease (~6-9 weeks post-transfer).

(A) Serum cytokines from *Rag1*$^{-/-}$ mice transferred as above and untransferred *Rag1*$^{-/-}$ controls (grey bar) determined by bender flow cytomix assay.

(B) Weight loss shown as percentage of original weight at day 0.

Data represent the pooled results of two independent experiments, bar represents the mean ± SEM, statistical significance determined using a Mann Whitney test.

Figure S2

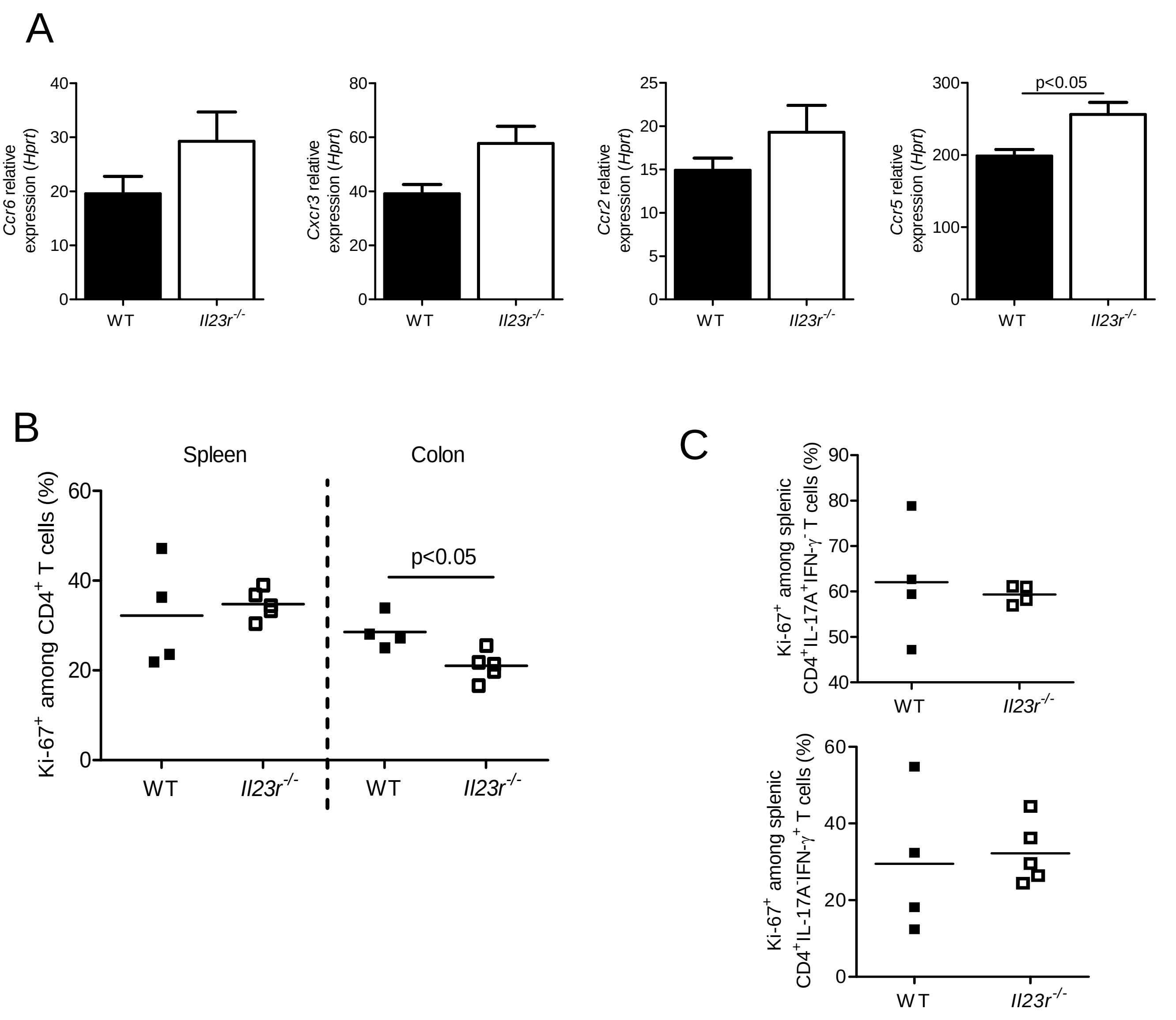

Figure S2. *Il23r*$^{-/-}$ T cells do not Display Defective Chemokine Receptor Expression or Homeostatic Expansion

C57BL.6.*Rag1*$^{-/-}$ mice were transferred with 4 x 10^5 CD4$^+$CD45RB^{hi} T cells from WT or *Il23r*$^{-/-}$ donors and sacrificed when recipients of WT T cells developed clinical signs of disease (~6 weeks post-transfer). IL-17A, IFN-γ and Ki-67 levels in T cells from the spleen and colon were assessed by intracellular FACS following *in vitro* re-stimulation with PMA and ionomycin.

(A) Chemokine receptor gene expression relative to *Hprt*

(B) Frequencies of Ki-67$^+$ cells among CD4$^+$ T cells in the spleen and colon.

(C) Frequencies of Ki-67$^+$ cells among IL-17A$^+$ or IFN-γ$^+$ CD4$^+$ T cells in the spleen.

Data represent the results of a single experiment, bar represents the mean ± SEM, and statistical significance determined using a Mann Whitney test

Figure S3

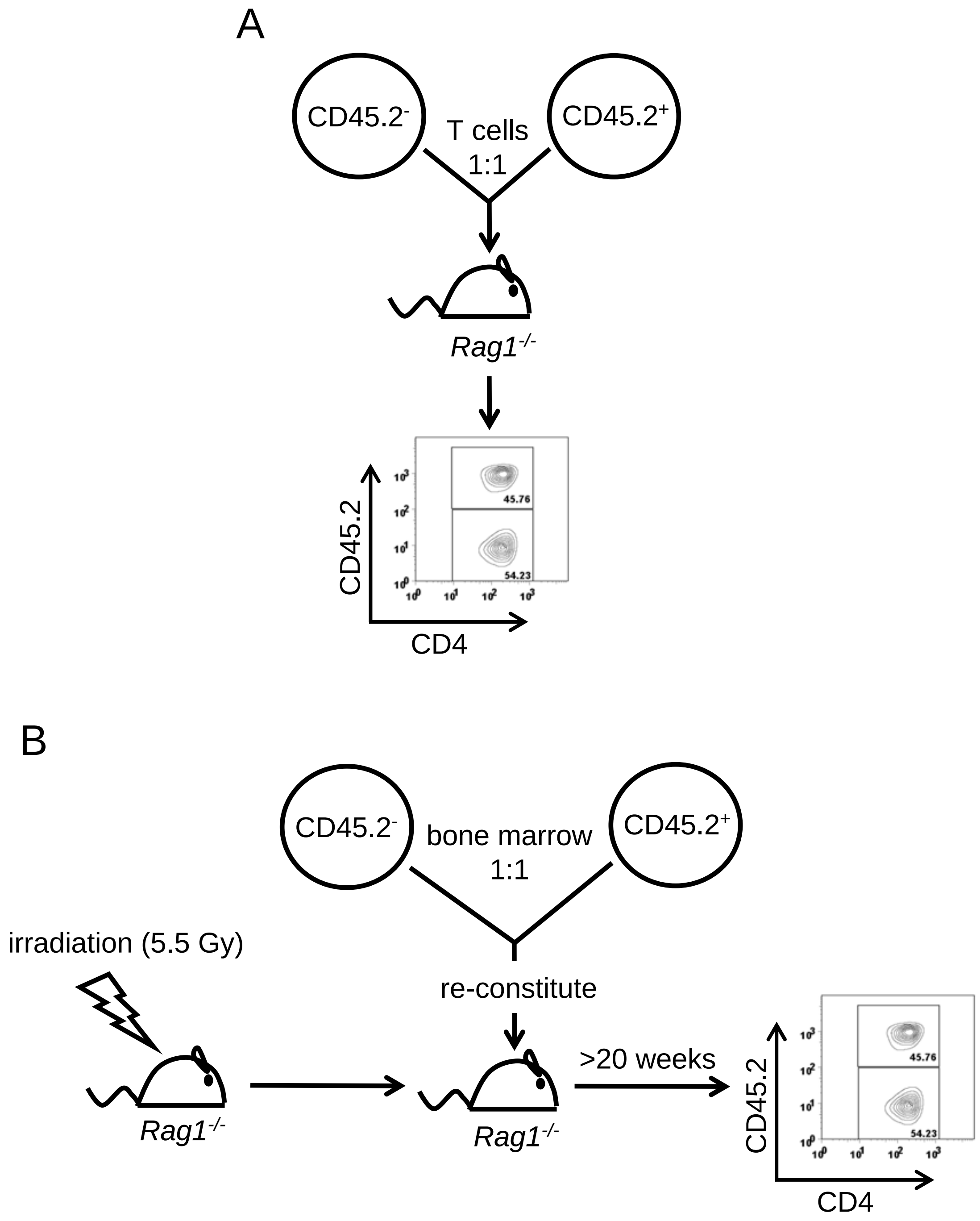

Figure S3. Co-Transfer of T cells and Generation of Bone Marrow Chimeras

(A) C57BL.6.*Rag1*$^{-/-}$ mice were transferred with 1:1 mixtures of CD45.2^{-} (WT) + CD45.2^{+} (WT) or CD45.2^{-} (WT) + CD45.2^{+}(*Il23r*$^{-/-}$) CD4^{+}CD45RB^{hi} T cells.

(B) Sublethally irradiated C57BL.6.*Rag1*$^{-/-}$ mice were reconstituted with 1:1 mixtures of CD45.2^{-} (WT) + CD45.2^{+} (WT) or CD45.2^{-} (WT) + CD45.2^{+}(*Il23r*$^{-/-}$) bone marrow cells. Chimeras were used in experiments >20 weeks post injection.

Figure S4

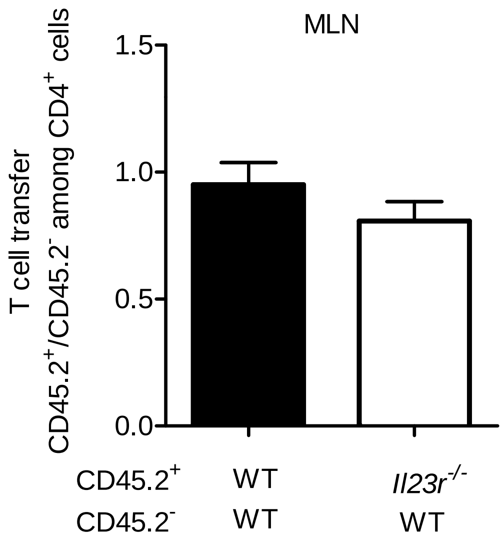

Figure S4. IL-23 Controls T cell Accumulation in the MLN via a Cell Extrinsic Mechanism

C57BL.6.*Rag1*$^{-/-}$ mice were transferred with 1:1 mixtures of CD45.2^{-} (WT) + CD45.2^{+} (WT) or CD45.2^{-} (WT) + CD45.2^{+}(*Il23r*$^{-/-}$) CD4^{+}CD45RB^{hi} T cells. Mice were sacrificed upon development of clinical signs of inflammation (~ 8 weeks) and populations of T cells were identified based on expression of CD45.2. The ratio of CD45.2^{+}/CD45.2^{-} T cells in the MLN is shown.

Data shown represent the pooled results from two independent experiments, bar represents the mean ± SEM, n=15 (WT), n=12 (*Il23r*$^{-/-}$). Statistical significance determined using a Mann Whitney test, differences were not significant.

Table S1. qPCR Primer Sets

Primer Set (PrimerBank ID)	Sequence (Forward & Reverse)	Probe (Dye Label)
Hprt	F: GACCGGTCCCGTCATGC<br>R: TCATAACCTGGTTCATCATCGC	ACCCGCAGTCCCAGCGTCGTC (FAM/VIC)
Il17a	F: GCTCCAGAAGGCCCTCAG<br>R: CTTTCCCTCCGCATTGACA	ACCTCAACCGTTCCACGTCACCCTG (FAM/TAMRA)
Il17f	F: GAGGATAACACTGTGAGAGTTGAC<br>R: GAGTTCATGGTGCTGTCTTCC	AGTTCCCCATGGGATTACAACATCACTC (FAM/TAMRA)
Il21	F: ATCCTGAACTTCTATCAGCTCCAC<br>R: GCATTTAGCTATGTGCTTCTGTTTC	AAGCCATCAAACCCTGGAAACAATAAGACA (FAM/TAMRA)
Il10	F: GCCACATGCTCCTAGAGCTG<br>R: CAGCTGGTCCTTTGTTTGAAA	CGGACTGCCTTCAGCCAGGTG (FAM/TAMRA)
Il22	F: GCTCAGCTCCTGTCACATCA<br>R: CACTGTCTCCTTCAGCCTTCT	GCGGTGACGACCAGAACATCCA (FAM/TAMRA)
Rorγt	F: CCGCTGAGAGGGCTTCAC<br>R: TGCAGGAGTAGGCCACATTACA	AAGGGCTTCTTCCGCCGCAGCCAGCAG (FAM/TAMRA)
Ccr6 (6753318a1)	F: CCTGGGCAACATTATGGTGGT<br>R: CAGAACGGTAGGGTGAGGACA	SYBR Green
Cxcr3 (6753458a1)	F: TACCTTGAGGTTAGTGAACGTCA<br>R: CGCTCTCGTTTTCCCCATAATC	SYBR Green
Ccr2 (6753466a1)	F: ATCCACGGCATACTATCAACATC<br>R: CAAGGCTCACCATCATCGTAG	SYBR Green
Ccr5 (31542356a1)	F: TTTTCAAGGGTCAGTTCCGAC<br>R: GGAAGACCATCATGTTACCCAC	SYBR Green

Supplemental Experimental Procedures

Assessment of Intestinal Inflammation

Mice were monitored regularly and sacrificed when symptoms of clinical disease (significant weight loss and/or diarrhoea) became apparent in control groups (T cell transfer), approximately ≥ 8 weeks after initiation of experiment. Mice infected with *H. hepaticus* and administered a blocking IL-10R mAb were sacrificed 4 weeks after infection (Kullberg et al., 2006). Samples of proximal, mid and distal colon were prepared as described (Izcue et al., 2008) and scored based on the following criteria: degree of epithelial hyperplasia and goblet cell depletion; leukocyte infiltration in lamina propria; area of tissue affected; and the presence of markers of severe inflammation (crypt abscesses, submucosal inflammation, ulcers). Sections were scored blindly by two independent researchers.

Bacteria

Helicobacter hepaticus NCI-Frederick isolate 1A (strain 51449; American Type Culture Collection) was grown in tryptic soy broth (Oxoid) supplemented with 10% fetal calf serum (PAA), containing trimethoprim, vancomycin, and polymixin B (all from Oxoid) under microaerophilic conditions as previously described (Hue et al., 2006). Bacterial viability was confirmed prior to infection using a bacterial live/dead kit (BacLight; Invitrogen), and mice infected orally with 5×10^7–1×10^8 CFU *H. hepaticus* on three consecutive days.

For induction of typhlocolits, *H. hepaticus*-infected mice were administered 1 mg of a blocking IL-10R mAb. (1B1.2) (O'Farrell et al., 1998), i.p., once a week, for a total of 4 weeks, commencing on day of first infection (Kullberg et al., 2006) Antibody was purified from hybridoma supernatant and shown to contain <1.0 endotoxin units/mg protein.
